# Etersalate prevents the formations of 6Aβ_16-22_ oligomer: An *in silico* study

**DOI:** 10.1371/journal.pone.0204026

**Published:** 2018-09-18

**Authors:** Son Tung Ngo, Xuan-Cuong Luu, Nguyen Thanh Nguyen, Van Van Vu, Huong Thi Thu Phung

**Affiliations:** 1 Computational Chemistry Research Group, Ton Duc Thang University, Ho Chi Minh City, Vietnam; 2 Faculty of Applied Sciences, Ton Duc Thang University, Ho Chi Minh City, Vietnam; 3 NTT Hi-Tech Institute, Nguyen Tat Thanh University, Ho Chi Minh City, Vietnam; 4 Department of Theoretical Physics, University of Science, Ho Chi Minh City, Vietnam; University of Minnesota Duluth, UNITED STATES

## Abstract

Oligomerization of amyloid beta (Aβ) peptides has been considered as the crucially causative agent in the development of Alzheimer's disease. Etersalate, a nonsteroidal anti-inflammatory oral drug (United State Food and Drug Administration—Unique Ingredient Identifier: 653GN04T2G) was previously suggested to bind well to proto-fibrils of Aβ peptides *in silico*. Here, the effect of etersalate on the oligomerization of soluble Aβ_16–22_ hexamer (6Aβ_16–22_) were extensively investigated using temperature replica exchange molecular dynamics (REMD) simulations over ~16.8 μs in total for 48 replicas (350 ns per replica). The results reveal that etersalate can enter the inner space or bind on the surface of 6Aβ_16–22_ conformations, which destabilizes the hexamer. Etersalate was predicted to able to cross the blood brain barrier using prediction of absorption, distribution, metabolism, and excretion—toxicity (preADMET) tools. Overall, although the investigation was performed with the low concentration of trial inhibitor, the obtained results indicate that etersalate is a potential drug candidate for AD through inhibiting formation of Aβ oligomers with the average binding free energy of -11.7 kcal/mol.

## Introduction

Fibrillation of amyloid beta peptides (Aβ) of 39–42 residues is found to be associated with Alzheimer’s disease (AD) [1–3]. Studies on Aβ fibrillation and its inhibition have drawn enormous interests since 4 decades ago. Later investigations led to the amyloid hypothesis, the widely accepted model for AD pathogenesis, in which Aβ oligomers also play various roles in injuring neurons [3, 4]. According to this hypothesis, Aβ oligomers can bind to receptors on the surface of cell membrane, activating microglia and astrocytes that causes progressive synaptic and neurotic injuries. Aβ oligomers can also direct effect on brain neurons’ synapses and neurocytes [5–9]. In addition, Aβ oligomers can insert themselves in the cell membrane, as well as mitochondria membrane, disrupting brain cell membrane and cellular ion homeostasis [10, 11]. Aβ oligomers are also found in the cytosol and within organelles, interfering with signalling pathways [12]. Moreover, Aβ oligomers can even spread between cells [13, 14]. Thus, inhibition of Aβ oligomerization has become an intriguing target for AD drug screening. Potential inhibitors, such as naproxen and curcumin, have been shown to alter the structures of Aβ dimers or interfere with oligomerization [15–17].

Aβ oligomers with higher numbers of monomers have been shown to be neurotoxic [4]. However, the structures of Aβ oligomers and the molecular details of their interactions with potential inhibitors have not been well understood because Aβ oligomers are intrinsically heterogeneous. Experimental studies of the inhibition of Aβ peptides oligomeric formation have been impeded [18] because Aβ oligomers exist transiently in a mixture consisting of different order of oligomers and fibrils [18, 19]. Computational studies have thus been instrumental in investigating Aβ oligomer systems [20–22]. The all-atom computational studies of full-length higher order Aβ oligomers require extremely large amounts of time for the computer to process because the folding time of Aβ peptides can last up to several hours [23]. Therefore, the short fragments of Aβ peptides are often used as a good model to evaluate the representative properties of the full-length one [24–27]. Accordingly, the hydrophobic core fragments of Aβ oligomers, such as Aβ_16–22_, Aβ_25–35_, and Aβ_30–36_, have been mainly chosen to be investigated [28–31]. Furthermore, Aβ_16–22_ fragment was often selected in designing the Aβ inhibitors [32–34], because the fragment forms fibril *in vitro* identified from the Aβ [35]. Results showed that these fragments well represented the self-assembly of Aβ oligomers and thus could be used to gain some important insights into the interactions of Aβ oligomers with their inhibitors.

Etersalate (also known as eterilate) is a nonsteroidal anti-inflammatory oral drug (NSAID) that was suggested to bind well to protofibrils of Aβ peptides *in silico* [36] due to having more than 80% of chemical similarity to curcumin, a potential inhibitor candidate of AD [16]. Although recent studies suggested that curcumin is a pan-assay interference compound [37] resulting in the failure of the clinical trial reported in 2012 [38], Transmission Electron Microscopy analysis indicated that the curcumin inhibited the self-assembly of the Aβ_40_ peptide [36]. Curcumin is currently tested in a long term AD therapy program [39], as well as in the prevention of the cognitive impairments in elders since 2017 [40]. Moreover, some nonsteroidal anti-inflammatory drugs were shown to be able to prevent Aβ oligomerization, such as celecoxib, ibuprofen, indomethacin, naproxen, nimesulide, and rofecoxib [41–46]. Thus, etersalate is a potential inhibitor for Aβ peptide oligomerization. The pharmacokinetics and pharmacology of etersalate are well-known except for its blood-brain barriers (BBB) crossing ability. Absorption, distribution, metabolism, excretion, and toxicity of etersalate have been well investigated previously (United State Food and Drug Administration—Unique Ingredient Identifier: 653GN04T2G). In this work, we evaluated the response of soluble Aβ_16–22_ hexamer system (6Aβ_16–22_) when etersalate was induced with low concentration of the inhibitor. Observed results indicated that etersalate is likely able to prevent the oligomerization process of 6Aβ_16–22_ in low concentration.

## Materials and methods

### Computational modeling

**Fig 1:**
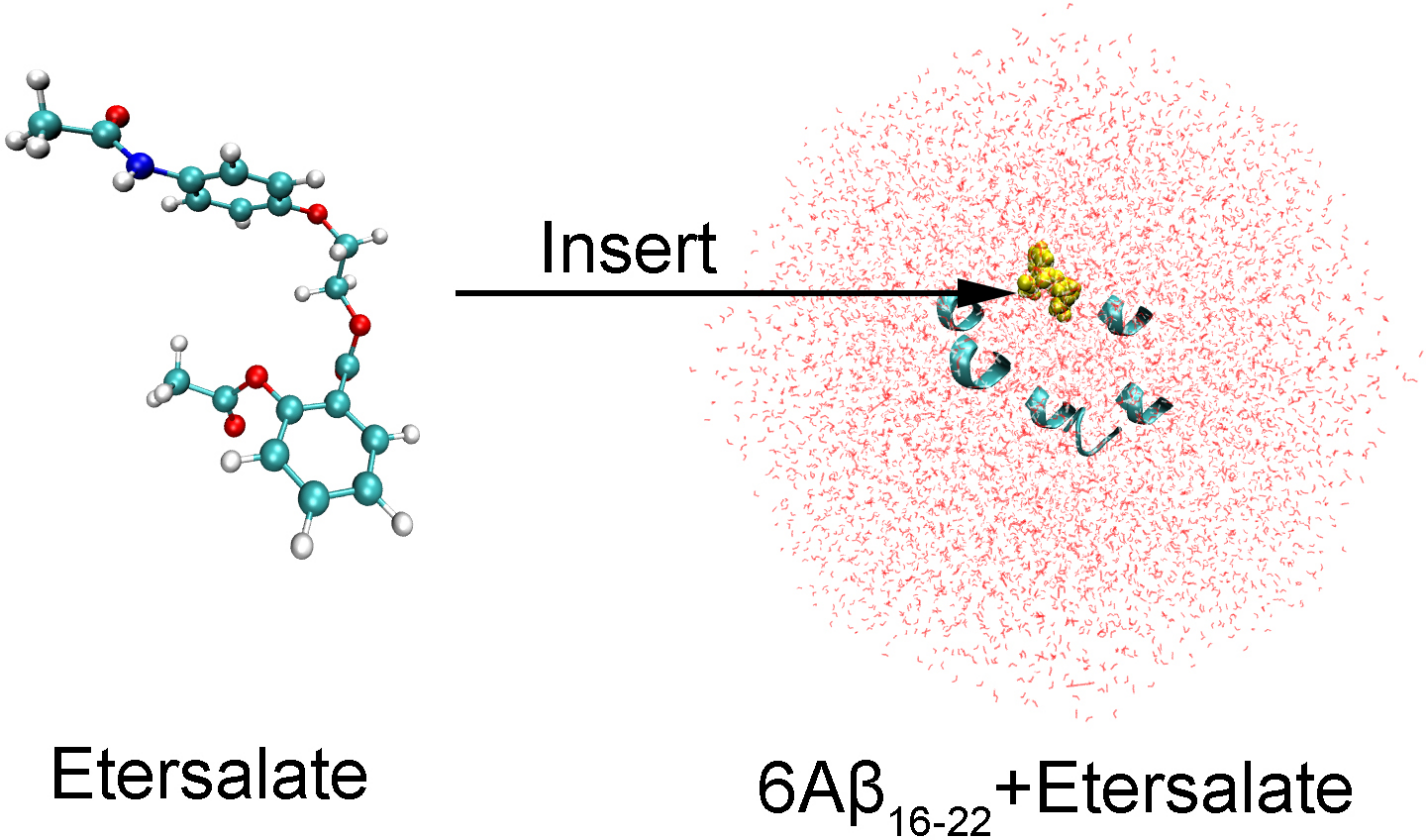
The 3-dimensional structure of etersalate and initial conformation of soluble 6Aβ_16–22_+etersalate in perspective view. The helical structure of Aβ_16–22_ peptide was randomly selected using the combination of PyMOL and VMD applications. Carbon, hydrogen, oxygen, and nitrogen atoms are shown in cyan, white, red, and blue, respectively.

The starting structure of 6Aβ_16–22_ was randomly set up using the combination of PyMOL [47] and Visual Molecular Dynamics (VMD) [48] protocols with the biased helical structure of each Aβ fragment (was mentioned in previous study [49]). Etersalate was randomly inserted into the soluble 6Aβ_16–22_ through VMD application (cf. Fig 1) [48]. Two systems were then solvated using TIP3P water model with a dodecahedron periodic boundary conditions [50]. Contemporaneously, etersalate was parameterized through general Amber force field with chemical quantum calculation at HF/6-31G(d,p) level [51]. The all-atoms force field Amber99SB-ILDN was employed to present the Aβ_16–22_ peptides [52]. The TIP3P water model [50] was served to solvate the system.

Two soluble systems were first minimized with steepest descent scheme. Then, the systems were simulated in canonical ensemble with the positional restraint non-solvated molecules condition. Finally, the temperature replica exchange molecular dynamics (T-REMD) simulations were applied to evaluate the effects of inhibitor on the hexamer system. The computational scheme was referred to the previous study [53]. There are 48 replicas with different temperatures ranging from 290 to 411 K (list of temperatures is described in S1 File). In accordance to previous works, the data was tended every 100 ps.

### Analysis and measurement

The system properties were estimated using the measurement of *C_α_* root mean square deviation (RMSD), radius of gyration (R_g_), solvent accessible surface area (SASA), and free energy surface (FES) using Groningen Machine for Chemical Simulations (GROMACS) tools [54]. The size of the soluble system was evaluated through the computation of collision cross section (CCS) with Ion Mobility Projection Approximation Calculation Tool (IMPACT) [55]. The secondary structure contents were determined with Define Secondary Structure of Proteins (DSSP) application [56]. The popular conformations were examined utilizing the clustering scheme of GROMACS [57]. In addition, the BBB crossing ability of the ligand was predicted using PreADMET server [58]. A sidechain contact of two residues is counted when the distance between their sidechain is smaller than 0.45 nm.

The absolute binding affinity between etersalate and 6Aβ_16–22_ was determined using the double-annihilation binding free energy method [59]. Detailed computational scheme was described in previous study [60], in which ten values of coupling parameter λ were used to eradicate the electrostatic interaction between an inhibitor and encompassing molecules including 0.0, 0.10, 0.25, 0.40, 0.55, 0.65, 0.75, 0.85, 0.95, and 1.00. Ten values of coupling parameter λ were assented to alter the van der Waals (vdW) interaction, including 0.0, 0.10, 0.20, 0.25, 0.30, 0.40, 0.55, 0.70, 0.85, and 1.00. The free energy difference between two states λ_i_ and λ_i+1_ was assessed using Bennet’s acceptance ratio protocol [61]. The free energy of binding between a monomer to the others was also measured using the same scheme.

## Results and discussion

### Predicted blood-brain barrier crossing ability of etersalate

Etersalate is an approved anti-inflammatory drug and its pharmacokinetics and pharmacology including absorption, distribution, metabolism, excretion, and toxicity have been well-known [62]. BBB crossing ability, which is expressed as log(BBB) is an important factor of neurodegenerative drugs. If a highly efficient inhibitor of Aβ oligomerization could not cross BBB, it could not be used as a drug for AD therapy. The PreADMET protocol has been used to successfully predict log(BBB) of Aβ oligomerization inhibitor candidates [63]. This protocol was applied to estimate the log(BBB) of etersalate. The predicted log(BBB) of etersalate is -1.36. This value falls in the range of log (BBB) (-2 to +1) of compounds capable of crossing BBB [58]. Etersalate is thus likely to be able to cross BBB and is an appropriate candidate for further consideration of Aβ oligomerization inhibition.

### REMD simulations of soluble 6Aβ_16–22_+etersalate

In order to get better sampling than conventional MD simulations, temperature REMD simulations were executed for solvated 6Aβ_16–22_+etersalate system. The computation length was 350 ns per replica, and the total simulation time was 16,800 ns with mean exchange rate of ~29%. The simulations were converged after approximately 250 ns of REMD simulations. The computed metrics were analysed over the simulation interval 250–350 ns at 299.2 K. The superposition of the computed values in different time intervals of simulations suggest the convergence of the simulations (S1 Fig). The differences of 6Aβ_16–22_+etersalate metrics to the isolated 6Aβ_16–22_, which was reported in previous study [34], provide the physical insights into the influence of ligand to the self-assembly of Aβ peptide.

**Fig 2:**
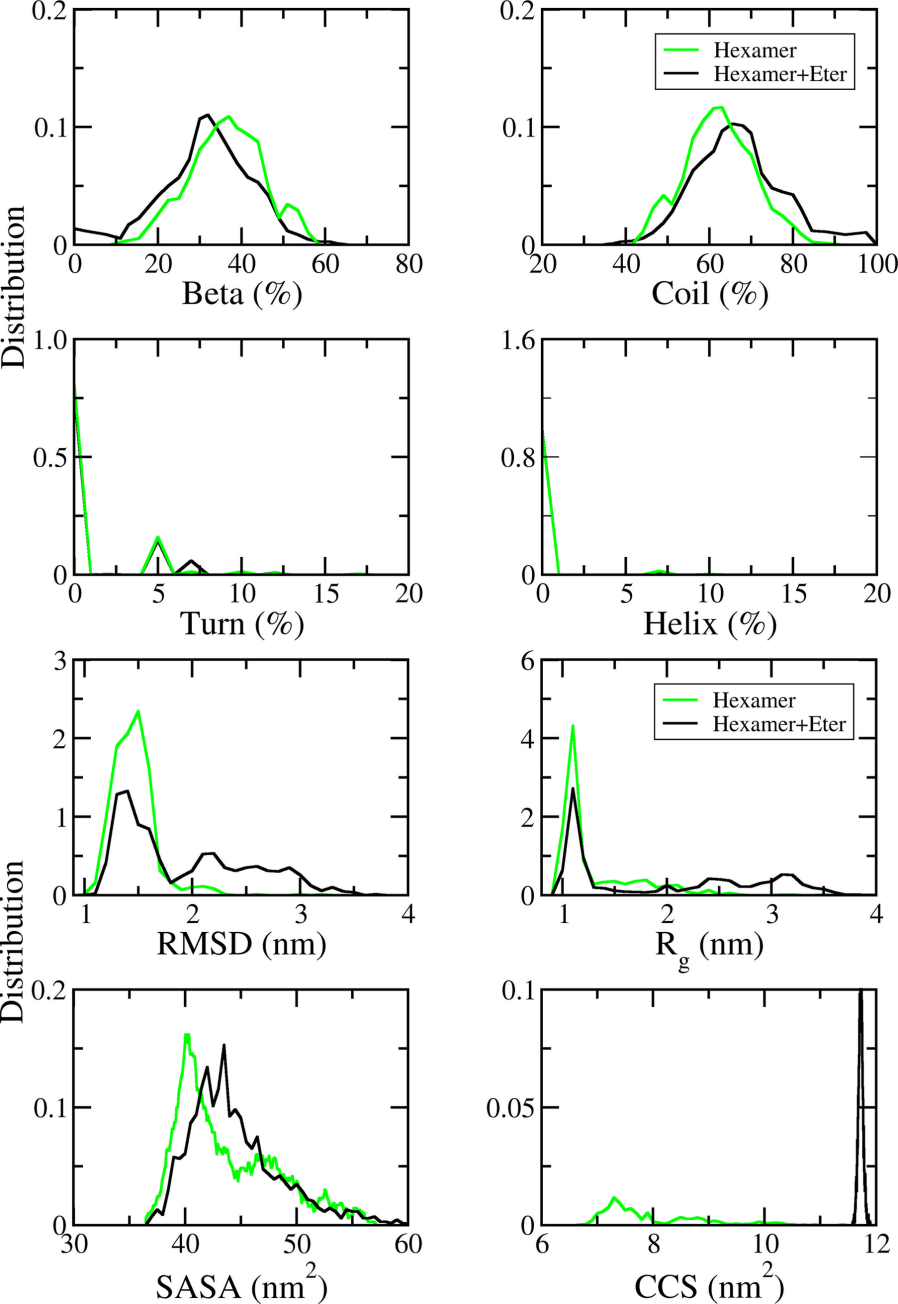
The distributions of measured values of the solvated 6Aβ_16–22_ (green lines) and 6Aβ_16–22_+etersalate (black lines) systems. The results were analysed from the REMD simulations in time window 250–350 ns at 299.2 K. The data of 6Aβ_16–22_ was reproduced from in our published results [34] with permission.

The secondary structure of the soluble hexamer was analysed in the last 100 ns of REMD simulations at 299.2 K utilizing the DSSP protocol [56]. In the presence of etersalate, the secondary structure terms of the hexamer system shift sizably (Fig 2). The β-content decreases by 4% from 36 ± 9% to 32 ± 10%. In contrast, coil content increases by 4% from 62 ± 8% to 66 ± 10%. Despite the large errors of the mean values, the shifts in β and coil contents are significant as demonstrated by the shift of the distribution curves shown in Fig 2. Furthermore, these significant differences (p < 0.05) are confirmed by statistical hypothesis testing results (z score calculations). The helix content also slightly increases (~0.3%), while the turn content remains unchanged. The tiny increase in the helix content is consistent with the increasing amount of free monomers in the system [64–66].

In the average of all considered snapshots during the last 100 ns of REMD simulations at 299.2 K, etersalate increases the size and dynamics of 6Aβ_16–22_ (Fig 2). In particular, *C*_*α*_ RMSD referred to the initial structure of 6Aβ_16–22_ (Fig 1A of [34]) is 1.46 ± 0.21 nm, which is significantly smaller than that of 6Aβ_16–22_+etersalate (1.93 ± 0.60 nm). In Fig 2, the distribution of RMSD in 6Aβ_16–22_ exhibits a narrow peak centred at 1.51 nm while in 6Aβ_16–22_+etersalate, this peak still presents but at ~45% lower magnitude. The conformations of 6Aβ_16–22_+etersalate with RMSD > 1.75 nm appears and accounts for ~48% of the total population.

The dimensions of the solvated systems are described through R_g_. The average *R*_*g*_ value increases from 1.30 ± 0.38 nm in 6Aβ_16–22_ to 1.98 ± 0.89 in 6Aβ_16–22_+etersalate. Approximately 82% of 6Aβ_16–22_ is distributed around the R_g_ peak at ~1.11 nm, and the rest disperses from ~1.60–2.50 nm (Fig 2). In 6Aβ_16–22_+etersalate, these two features decreases by ~ 34%, while a new population appears in the R_g_ range of 1.5–4 nm and accounts for ~53% of the total population. It is noted that in the conformations with R_g_ > 2 nm, at least one monomer has no contact with the others. The shift in R_g_ indicates that etersalate can induce significant dissociation of the hexamer. Consistent with changes in RMSD and R_g_ distributions, SASA and CCS also increase in the presence of etersalate. SASA slightly increases from 43.88 ± 4.52 nm^2^ to 44.52 ± 4.18 nm^2^. Notably, CCS increases from 7.94 ± 0.89 nm^2^ to 11.73 ± 0.42 nm^2^. CSS distribution exhibits a remarkable shift from a widespread feature between 6.5–11 nm^2^ to a narrow peak at 11.73 nm^2^ (Fig 2).

Recent studies on the effect of graphene oxide nanosheets and fullerenes indicated that larger compounds have stronger inhibitory effect on the self-aggregation of Aβ peptides [32, 67]. However, size does not appear to be the controlling factors in clinically relevant compounds. Etersalate (357 g/mol) and propafenone (341 g/mol) are more than 80% chemically similar. However, while etersalate reduces more than 12% of the total amount of β-structure of 6Aβ_16–22_ (from 36 to 32%) as shown in this work, propafenone was previously shown to have no effect on 6Aβ_16–22_ [34]. In addition, although C60 is significantly larger than etersalate, the effect of C60 on β-content of Aβ_16–22_ system [32] is significantly less than that of etersalate. Overall, it is more likely that the chemical nature of etersalate, but not its size, contribute the most to its inhibitory effect on the self-aggregation of Aβ_16–22_ peptides.

### Effects of etersalate on the distribution of secondary structure of soluble 6Aβ_16–22_ per residue

**Fig 3:**
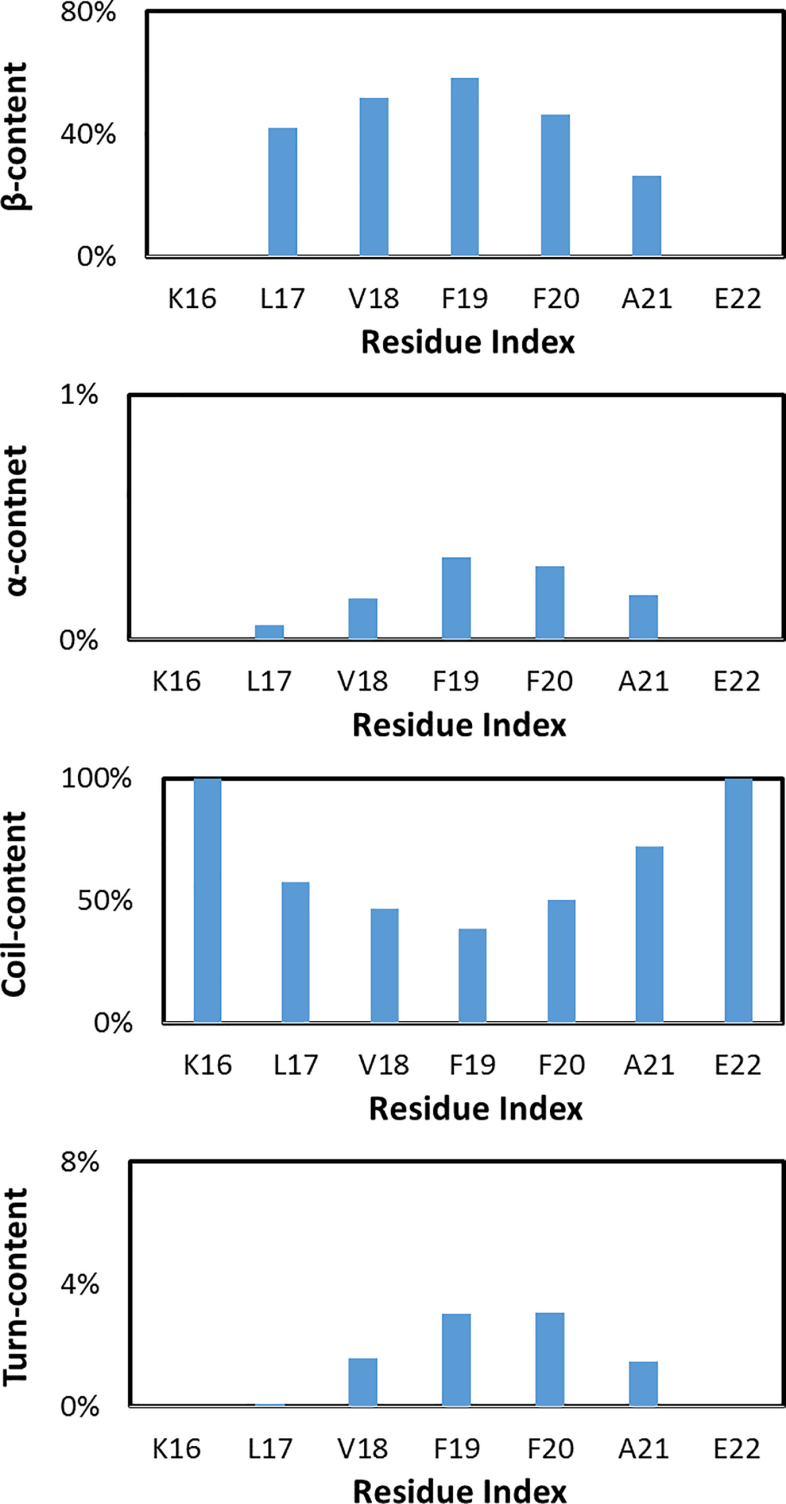
The secondary structure terms of 6Aβ_16–22_+etersalate.

The distributions of β, coil, turn, and α contents per residues averaged for all six chains in soluble 6Aβ_16–22_+etersalate are shown in Fig 3. Overall, as mentioned above, the distributions of β-content and coil structure of the 6Aβ_16–22_ are significantly different in present of the inhibitor. The β, α and turn content are dominant in the middle of the peptide chains, while coil content spreads throughout the chain with 100% at the two ends of peptide (residues K16 and E22). Consequently, there is no β, α and turn content in these end-residues. The β-content of solvated 6Aβ_16–22_ per residue reduces about 30% in the presence of etersalate (from 65% to 41% at residue F19), while α-content dramatically increases two to four fold (from 0.47% to 1.86% at residue F19). Especially, α-content in residue L17 jumps from zero to around 0.9% when etersalate presents. Both coil and turn contents in 6Aβ_16–22_+etersalate increase compared to that in 6Aβ_16–22_, especially 3 times higher in turn content at residues F19 (from 2% to 6%).

### Inter-chain contacts

The intermolecular contacts between constituting monomer of 6Aβ_16–22_+etersalate were examined to evaluate the physical effects of etersalate on the nature of binding between isolated chains of 6Aβ_16–22_. The intermolecular contacts included the sidechain contacts that were determined as described in the Materials & methods section. These values were probed every 100 ps over the last 100 ns of REMD simulation at 299.2 K.

**Fig 4:**
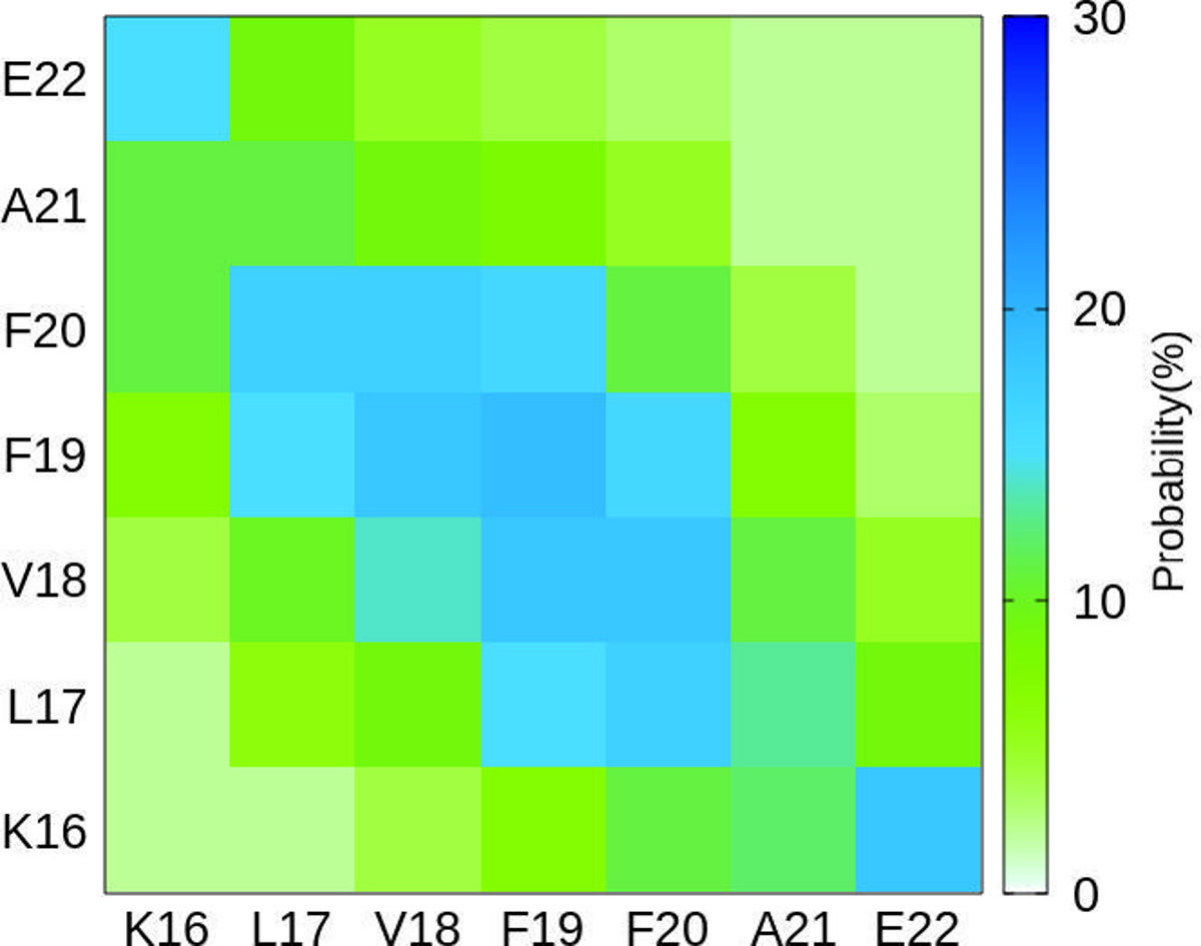
The sidechain contact maps between the constituting chains of 6Aβ_16–22_+etersalate.

The sidechain contacts between different heavy atoms of different chains were measured and averaged over the considered snapshots. The sidechain contact maps were then created based on the probability of these values (Fig 4). The difference between the metrics of 6Aβ_16–22_+etersalate and isolated 6Aβ_16–22_ [34] are observed. In qualitative, chains in two systems contact to each other in an anti-paralleled fashion with the dominant interactions reside in the middle from residues L17 to F20. This is in consistent with the high β-content in the middle part of chains in two cases. However, in quantitative, the overall sidechain contact strength significantly reduces in 6Aβ_16–22_+etersalate, particularly at the middle-chain interaction which is corresponding to the reduction of β-content and marked increase in α and turn contents in comparison to that in isolated 6Aβ_16–22_ [34]. In addition, significant contacts between residues at N- and C-terminal ends (K16 and E22) of anti-paralleled chains are also observed, indicating that the computational sampling was adequate. Overall, the mean sidechain contacts of 6Aβ_16–22_ (11.8%) [34] are significantly decreased to 9.2% when etersalate is induced. The decrease is much larger than the case when propafenone is induced (~11.5% in previous study [34]). The lower number of sidechain contacts in 6Aβ_16–22_+etersalate is in good agreement with the observation that etersalate can reduce the integrity of 6Aβ_16–22_ structure.

### Etersalate alters the popular structures of the hexamer

**Fig 5:**
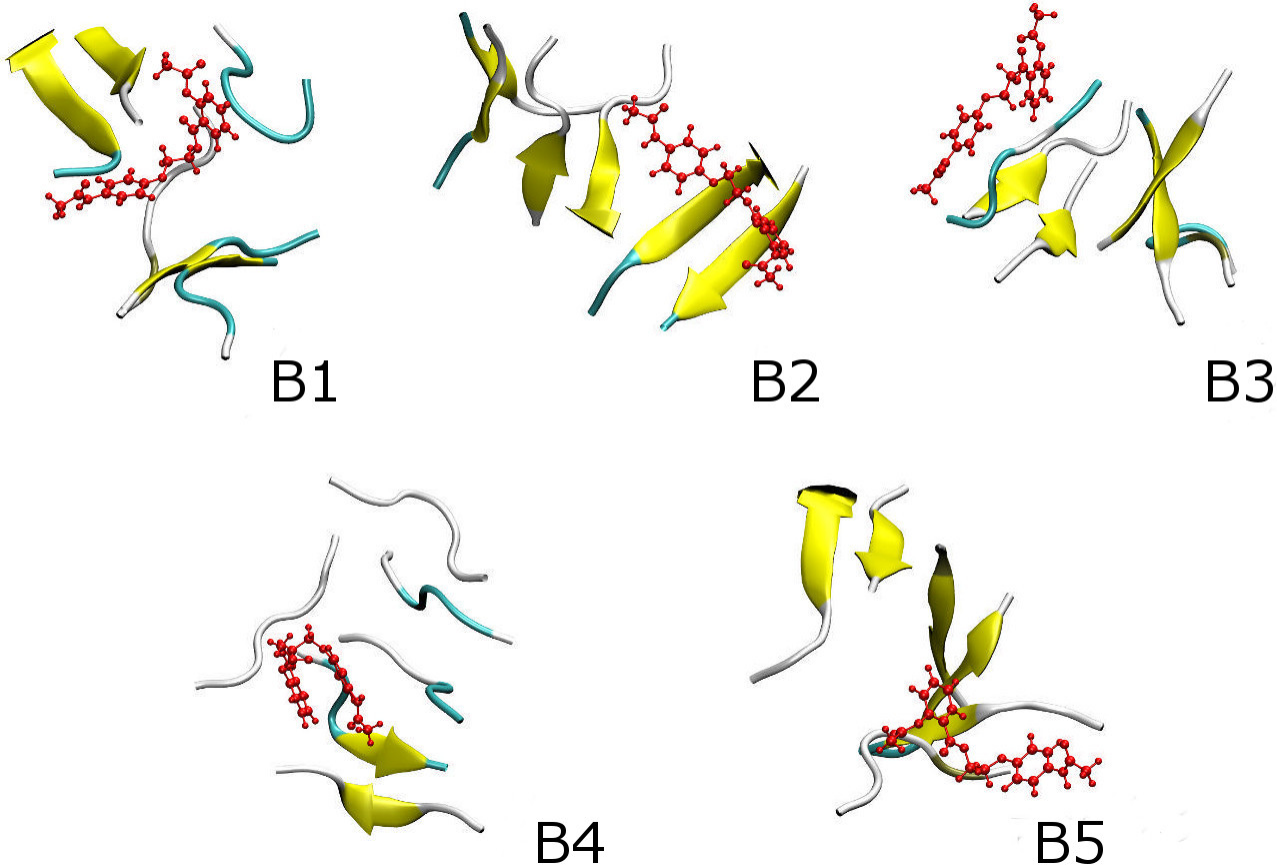
Popular conformations obtained by clustering method of the solvated 6Aβ_16–22_+etersalate system (B1-B5). Etersalate is shown in red.

In order to better picture the effect of etersalate on the hexamer, we analyzed the popular structures of 6Aβ_16–22_+etersalate obtained using *C*_*α*_ RMSD clustering method with a cutoff of 0.3 nm. More than 660 clusters of the 6Aβ_16–22_+etersalate systems were counted in comparison with 300 of the isolated 6Aβ_16–22_. The fewer number of clusters may imply the faster aggregation process. Five of the most populated conformations of 6Aβ_16–22_+etersalate (denoted as **B1-B5**) are shown Fig 5. The total population of **B1-B5** is 9.9% of all equilibrium snapshots, which is approximately 3 times smaller than that of isolated 6Aβ_16–22_ (27.2%).

The secondary structure metrics of **B1-B5** structures are shown in Table 1. In average, 6Aβ_16–22_+etersalate contains only 35% β-structure (versus with 41% of isolated 6Aβ_16–22_). **B5** and **B4** contain 48% and 14% β-structure, respectively, which are the most and the least among the representatives of 6Aβ_16–22_+etersalate. The corresponding metrics of isolated 6Aβ_16–22_ were range from 36 to 52%. Etersalate thus appears to induce fluctuation in the secondary structure of the hexamer.

**Table 1:** The secondary structure terms and CCS of the popular structures of 6Aβ_16–22_+etersalate (B1-B5), which were predicted using the DSSP and IMPACT tools.

Conformation	Beta content (%)	Coil content (%)	Turn content (%)	Helix content (%)	Population (%)	CCS (nm^2^)
B1	36	64	0	0	3.5	9.14
B2	45	55	0	0	2.0	9.35
B3	31	64	5	0	1.7	8.85
B4	14	81	5	0	1.5	8.58
B5	48	52	0	0	1.2	9.26
**Average**	**35**	**63**	**2**	**0**	**2.0**	**9.04**

Etersalate is observed in the center of **B1** (Fig 5), the Aβ pattern is thus restricted. In **B2-B5**, the inhibitor appends on the surface and destabilizes the oligomers. The population of representative structures of 6Aβ_16–22_+etersalate is thus much decreased compared to 6Aβ_16–22_ as mentioned above. The increase of CCS was observed from 8.60 nm (isolated 6Aβ_16–22_) to 9.04 nm (6Aβ_16–22_+etersalate) that is in good agreement with the whole trajectory analysis described above.

### Stable structures obtained from combination of the FES and clustering methods

**Fig 6:**
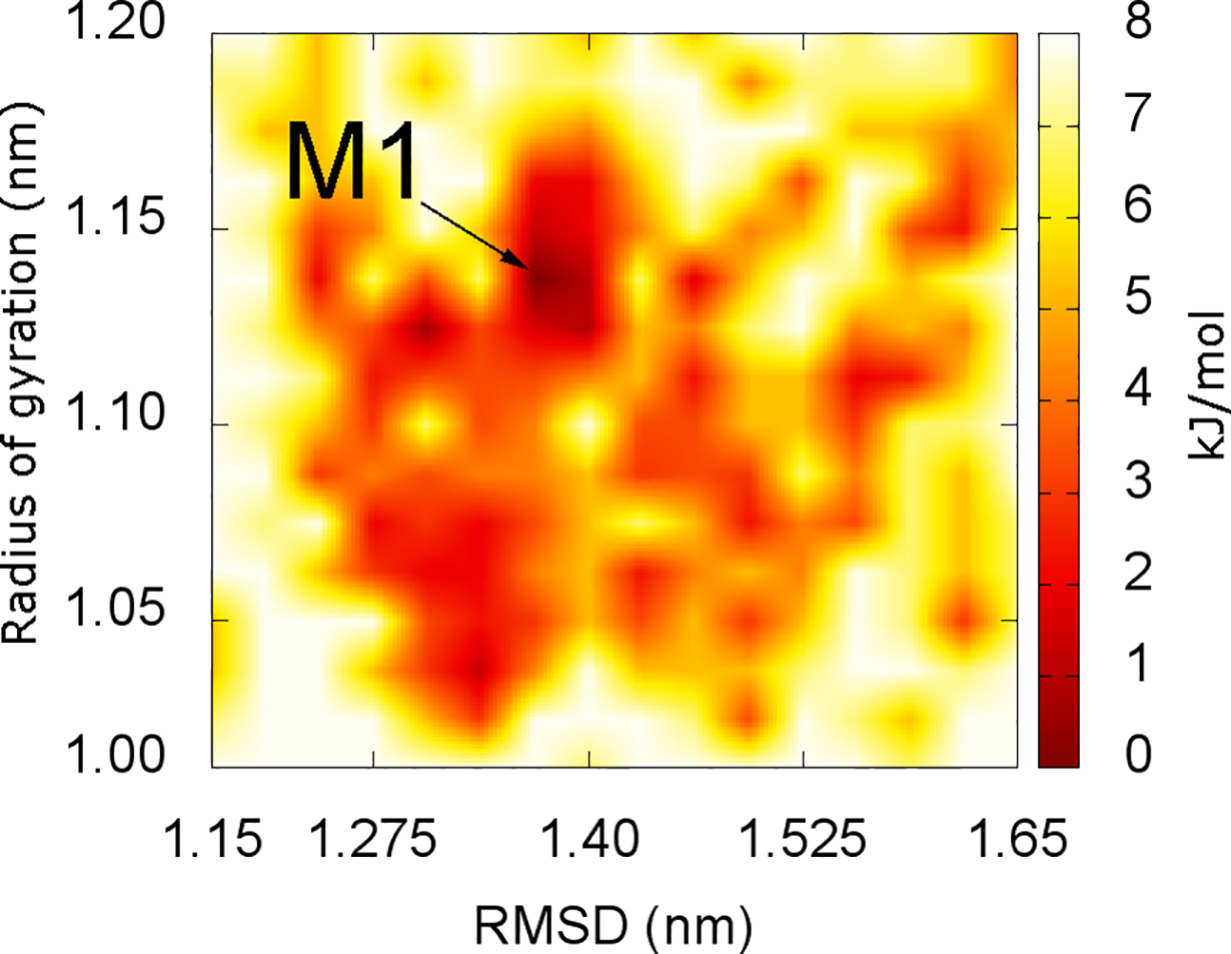
The free energy landscape of 6Aβ_16–22_+etersalate. The global minima of soluble system was found to be (1.35, 1.13) in comparison with (1.06, 1.60) of 6Aβ_16–22_, which was mentioned in previous results [34].

The representative structures of 6Aβ_16–22_+etersalate were determined using the combination of FES and clustering methods referring previous study [68]. This combination has been proven to be markedly suitable in studying the stable structures of β-amyloid systems [69, 70]. FES was constructed with *C_α_* RMSD and *R*_*g*_ coordinates using the GROMACS tool “gmx sham” [71]. The result is shown in Fig 6. RMSD and *R*_*g*_ are in range of 1.15–1.65 nm.

The minimum of soluble system was found to be **M1** (1.35, 1.13) (Fig 6) compared to (1.06, 1.60) of 6Aβ_16–22_ as described in our previous study [34]. **M1** conformation matches **B1** structure in clustering study (Fig 5). The representative structure of 6Aβ_16–22_+etersalate (**B1**) is significantly different from that of the 6Aβ_16–22_ (noted as **A1’** in Fig 4 of reference [34]).

As described in Table 1, **M1** (**B1**) population appears around 3.5% (with *C*_*α*_ RMSD cutoff of 0.3 nm) with reduced β-structure (36%) and increased coil-structure (64%) compared to **A1’** of 6Aβ_16–22_ (7.8%) with 52% of β-structure and 48% of coil-structure [34]. Etersalate is found in the middle of 6Aβ_16–22_ bundle (Fig 5). These results indicate that etersalate can get into the core of 6Aβ_16–22_ which loosens the structure of hexamer, and reduce beta content and lower number of sidechain contacts.

### Binding free energy between the constituting monomers to others

The determination of binding free energy also provides the information on the nature of the oligomeric formation [20]. The free energy perturbation method is known one of the most accurate methods until now. The double-annihilation binding free energy scheme was thus applied to investigate the nature of binding between the constituting monomers of soluble 6Aβ_16–22_ and 6Aβ_16–22_+etersalate systems. The calculations were applied on the conformation **B1** (Fig 5) and **A1’** (Fig 5 of ref. [34]). In this scheme, the free energy difference of binding between isolated chains to the others was evaluated. The results were averaged over the six monomers in each system.

The free electrostatic energy Δ*G*_*elec*_ of 6Aβ_16–22_ is -42.01 kcal/mol which is much larger than that in 6Aβ_16–22_+etersalate (-29.09 kcal/mol). Meanwhile, the free vdW interaction energy Δ*G*_*vdW*_ in 6Aβ_16–22_+etersalate is significantly increased (-20.10 kcal/mol) in comparison to that in 6Aβ_16–22_ (-7.50 kcal/mol). Overall, a constituting chain formed approximately -49.18 ± 3.00 kcal/mol of binding free energy to the other monomers in 6Aβ_16–22_+etersalate which is similar to that in 6Aβ_16–22_ (-49.51 +/- 2.95 kcal/mol). These results indicate that the monomers in 6Aβ_16–22_ bind to each other mainly due to the electrostatic interactions which are considered as a “near” binding force. The observation is different to the intermolecular contacts analysis above because the sidechain contacts were measured entire simulation space. However, the binding free energy calculation was carried out only for optimized structure of the hexamer systems. It may argue that all equilibrium snapshot should be considered in the computer-aided drug design problem instead of the optimized structure only.

### Absolute binding affinity of etersalate to Aβ hexamer

The absolute binding affinity is a major metric of computer-aided drug design due to its association to experimental inhibition constant. In this work, the binding free energy (Δ*G*_*FEP*_) of the etersalate to 6Aβ_16–22_ peptides was evaluated using representative structures of the soluble complex obtaining above as initial conformations. Twenty values of coupling parameter λ were used to annihilate the etersalate from both soluble complex and isolated ligand systems. The different work of these processes provides the difference in Gibbs free energy of binding between 6Aβ_16–22_ and etersalate. Δ*G*_*FEP*_ is the sum of coulomb (Δ*G*_*cou*_) and vdW (Δ*G*_*vdW*_) interactions. The length of each estimation is 1 ns, in which the free energy is obtained from 500–1000 ps to evade the initial variation caused by modification of the Hamiltonian interaction. Observed results are shown in Table 2. In particular, the etersalate adopts a slightly smaller the binding free energy (-19.7 kcal/mol) to 6Aβ_16–22_ compared with EGCG (-25.4 kcal/mol) [49] when the optimized structures (shape **M1** or **B1**) were considered only. However, as mentioned above, the interacted picture of ligand to peptide may not be cleared when the optimized structures were examined only. The free energy estimation was also carried out for five representative conformations, which was produced from clustering method. In which, etersalate forms the strongest non-bonded contact to the **B1** and **B2** conformations with Δ*G*_*FEP*_ of -19.7 and -19.8 kcal/mol, respectively. The worst affinity (-3.7 kcal/mol) is observed with **B4** conformation. Results in Table 2 also show that vdW interactions are the major components of the binding between etersalate and 6Aβ_16–22_. The population-averaged Δ*G*_*FEP*_ for all five representative structures is -13.8 kcal/mol. This metric is quite larger than the values of naproxen and ibuprofen (other NSAIDs) using MM-PBSA method (-9.45 and -8.31 kcal/mol, respectively) [16].

**Table 2:** The binding affinity of etersalate to 6Aβ_16–22_ peptides obtained with double-annihilation binding free energy method. All metrics are in kcal/mol unit.

Conformation	Δ*G*_*cou*_	Δ*G*_*vdW*_	Δ*G*_*FEP*_
**B1**	2.26	-21.96	-19.70 ± 1.28
**B2**	-7.24	-12.60	-19.84 ± 1.88
**B3**	-5.28	-3.60	-8.88 ± 1.03
**B4**	2.33	-6.03	-3.70 ± 1.17
**B5**	-1.26	-5.19	-6.44 ± 1.29

In addition, as we attained above and compared to previous study [49], the formations of 6Aβ_16–22_ is larger affected upon appearance of etersalate than EGCG, although the EGCG forms a larger binding free energy. Though the binding free energy is a critical factor of computer-aided drug design problem in general due to relation to experimental inhibition constant, various metrics should be considered in designing drug for Aβ oligomer including sidechain contacts, secondary structure, population of clusters, CCS, etc. The obtained picture would thus generalize.

## Conclusion

As mention above, the hydrophobic core fragment of Aβ oligomers Aβ_16–22_ was often selected in designing inhibitors of the self-assembly of Aβ peptides because the fragment forms fibril *in vitro* identified from the Aβ [35]. The formation of 6Aβ_16–22_+etersalate was monitored in comparison with isolated 6Aβ_16–22_ system [34] through intensive REMD simulations. The short sequence forms the anti-parallel state of β-strands that is in good agreement with previous studies [34, 72].

Observed results indicate that etersalate, a current NSAID, is a highly potential inhibitor of the Aβ oligomers as celecoxib, ibuprofen, indomethacin, naproxen, nimesulide, and rofecoxib [41–46]. The presence of etersalate forces the beta content of the soluble hexamer to decrease, concomitantly with the increase in the coil content. The inhibitor can enter the inner space between the monomers of 6Aβ_16–22_ or bind to the surface of the hexamer conformations, which destabilizes the hexamer structure. Rigorous analysis of RMSD, Rg, SASA, and CCS indicate that etersalate binding leads to significant changes in the conformations and dynamics of the hexamer. Notably, in the presence of etersalate at least one monomer of a significant fraction of the hexamer dissociates and forms no contact with other monomers. Thus, etersalate is a potentially and highly efficient inhibitor of Aβ oligomerization, although the binding free energy between etersalate and Aβ is moderate with an average value of -11.7 kcal/mol. In addition, this compound is predicted to be able to permeate from blood vessel into the brain. Thus, etersalate is a potential drug candidate for AD therapy. Further *in vitro* and/or *in vivo* investigations are anticipated in evaluating etersalate as a drug for AD.

## Supporting information

S1 Fig The distributions of radius of gyration (Rg), RMSD, CCS, and SASA of the solvated 6Aβ_16–22_+etersalate system in different computational time intervals 250–320 ns (red dotted lines), 280–350 ns (blue dotted lines), 270–340 ns (yellow dotted lines), and 250–350 ns (black curves). Observed results indicate that the computations are converged. The results were then analysed from the REMD simulations in time window 250–350 ns at 299.2 K.
(DOCX)

S2 Fig The diffusion entire temperature space of the 1^st^ replica monitoring over intervals 300–350 ns of REMD simulations. (DOCX)

S1 File (DOCX)
