# Supplementary material for: Etersalate prevents the formations of 6Aβ16-22 oligomer: An in silico study
Source: PLoS One. 2018 Sep 18;13(9):e0204026. doi: 10.1371/journal.pone.0204026 (PMC6143259; doi:10.1371/journal.pone.0204026)
Supplement: S1 Fig — Observed results indicate that the computations are converged. The results were then analysed from the REMD simulations in time window 250–350 ns at 299.2 K. (DOCX) [file pone.0204026.s001.docx]

Etersalate prevents the formations of 6Aβ_16-22_ oligomer: an in silico study

*Short title*: Etersalate effects on structure of 6Aβ_16-22_ oligomer

Son Tung Ngo^1,2*^, Xuan-Cuong Luu^3^, Nguyen Thanh Nguyen^4^, Van Van Vu^3^and HUONG THI THU PHUNG^3*^

^1^ Computational Chemistry Research Group, Ton Duc Thang University, Ho Chi Minh City, Vietnam

^2^ Faculty of Applied Sciences, Ton Duc Thang University, Ho Chi Minh City, Vietnam

^3^ NTT Hi-Tech Institute, Nguyen Tat Thanh University, Ho Chi Minh City, Vietnam

^4^ Department of Theoretical Physics, University of Science, Ho Chi Minh City, Vietnam

* Corresponding author

E-mail*:* ngosontung@tdtu.edu.vn (STN); ptthuong@ntt.edu.vn (HTTP)





**S1 Fig: The distributions of radius of gyration (Rg), RMSD, CCS, and SASA of the solvated 6Aβ_16-22_+etersalate system in different computational time intervals 250-320 ns (red dotted lines), 280-350 ns (blue dotted lines), 270-340 ns (yellow dotted lines), and 250-350 ns (black curves)**. Observed results indicate that the computations are converged. The results were then analysed from the REMD simulations in time window 250-350 ns at 299.2 K.
