# Supplementary material for: Etersalate prevents the formations of 6Aβ16-22 oligomer: An in silico study
Source: PLoS One. 2018 Sep 18;13(9):e0204026. doi: 10.1371/journal.pone.0204026 (PMC6143259; doi:10.1371/journal.pone.0204026)
Supplement: S2 Fig — (DOCX) [file pone.0204026.s002.docx]

Etersalate prevents the formations of 6Aβ_16-22_ oligomer: an in silico study

*Short title*: Etersalate effects on structure of 6Aβ_16-22_ oligomer

Son Tung Ngo^1,2*^, Xuan-Cuong Luu^3^, Nguyen Thanh Nguyen^4^, Van Van Vu^3^and HUONG THI THU PHUNG^3*^

^1^ Computational Chemistry Research Group, Ton Duc Thang University, Ho Chi Minh City, Vietnam

^2^ Faculty of Applied Sciences, Ton Duc Thang University, Ho Chi Minh City, Vietnam

^3^ NTT Hi-Tech Institute, Nguyen Tat Thanh University, Ho Chi Minh City, Vietnam

^4^ Department of Theoretical Physics, University of Science, Ho Chi Minh City, Vietnam

* Corresponding author

E-mail*:* ngosontung@tdtu.edu.vn (STN); ptthuong@ntt.edu.vn (HTTP)





**S2 Fig. The diffusion entire temperature space of the 1^st^ replica monitoring over intervals 300-350 ns of REMD simulations.**
