# Supplementary material for: Etersalate prevents the formations of 6Aβ16-22 oligomer: An in silico study
Source: PLoS One. 2018 Sep 18;13(9):e0204026. doi: 10.1371/journal.pone.0204026 (PMC6143259; doi:10.1371/journal.pone.0204026)
Supplement: S1 File — (DOCX) [file pone.0204026.s003.docx]

Etersalate prevents the formations of 6Aβ_16-22_ oligomer: an in silico study

*Short title*: Etersalate effects on structure of 6Aβ_16-22_ oligomer

Son Tung Ngo^1,2*^, Xuan-Cuong Luu^3^, Nguyen Thanh Nguyen^4^, Van Van Vu^3^and HUONG THI THU PHUNG^3*^

^1^ Computational Chemistry Research Group, Ton Duc Thang University, Ho Chi Minh City, Vietnam

^2^ Faculty of Applied Sciences, Ton Duc Thang University, Ho Chi Minh City, Vietnam

^3^ NTT Hi-Tech Institute, Nguyen Tat Thanh University, Ho Chi Minh City, Vietnam

^4^ Department of Theoretical Physics, University of Science, Ho Chi Minh City, Vietnam

* Corresponding author

E-mail*:* ngosontung@tdtu.edu.vn (STN); ptthuong@ntt.edu.vn (HTTP)

The temperature REMD simulations were performed at 48 different temperatures involving 290, 292.38, 294.64, 296.91, 299.2, 301.5, 303.82, 306.14, 308.48, 310.83, 313.2, 315.58, 317.97, 320.37, 322.79, 325.23, 327.67, 330.13, 332.61, 335.1, 337.85, 340.37, 342.9, 345.44, 348, 350.57, 353.16, 355.77, 358.38, 361.01, 363.66, 366.32, 369, 371.68, 374.39, 377.11, 379.85, 382.6, 385.37, 388.16, 390.96, 393.77, 396.6, 399.45, 402.31, 405.19, 408.09, and 411.
